# Near-trench slip potential of megaquakes evaluated from fault properties and conditions

**DOI:** 10.1038/srep28184

**Published:** 2016-06-20

**Authors:** Tetsuro Hirono, Kenichi Tsuda, Wataru Tanikawa, Jean-Paul Ampuero, Bunichiro Shibazaki, Masataka Kinoshita, James J. Mori

**Affiliations:** 1 Department of Earth and Space Science, Graduate School of Science, Osaka University, Toyonaka, Osaka 560-0043, Japan; 2 Center for Safety and Reliability Engineering, Institute of Technology Shimizu Corporation, Koto, Tokyo 135-8530, Japan; 3 Kochi Institute for Core Sample Research, Japan Agency for Marine–Earth Science and Technology, Nankoku, Kochi 783-8502, Japan; 4 Division of Geological and Planetary Sciences, California Institute of Technology, Pasadena, CA 91125, USA; 5 International Institute of Seismology and Earthquake Engineering, Building Research Institute, Tsukuba, Ibaraki 305-0802, Japan; 6 Earthquake Research Institute, University of Tokyo, Bunkyo, Tokyo 113-0032, Japan; 7 Earthquake Hazards Division, Disaster Prevention Research Institute, Kyoto University, Uji, Kyoto 611-0011, Japan

## Abstract

Near-trench slip during large megathrust earthquakes (megaquakes) is an important factor in the generation of destructive tsunamis. We proposed a new approach to assessing the near-trench slip potential quantitatively by integrating laboratory-derived properties of fault materials and simulations of fault weakening and rupture propagation. Although the permeability of the sandy Nankai Trough materials are higher than that of the clayey materials from the Japan Trench, dynamic weakening by thermally pressurized fluid is greater at the Nankai Trough owing to higher friction, although initially overpressured fluid at the Nankai Trough restrains the fault weakening. Dynamic rupture simulations reproduced the large slip near the trench observed in the 2011 Tohoku-oki earthquake and predicted the possibility of a large slip of over 30 m for the impending megaquake at the Nankai Trough. Our integrative approach is applicable globally to subduction zones as a novel tool for the prediction of extreme tsunami-producing near-trench slip.

Fault rupture during the 2011 Tohoku-oki earthquake (M_w_ 9.0) nucleated at 17 km depth on the interface between the Pacific and the North American plates^1^ and reached the seafloor at the trench axis^2^ with very large coseismic displacement exceeding 50 m^3^,^4^. This earthquake has brought into focus the role of dynamic stresses in driving seismic slip in the shallow portion of subduction zones^5^, because shallow unconsolidated materials are often considered to behave aseismically^6^. Slip is fundamentally related to the stress drop on the fault^7^, which is primarily governed by the stress state and by dynamic mechanisms that weaken faults. Pressurization of the interstitial fluid by frictional heating (thermal pressurization^8^, TP), which is an important fault weakening mechanism^9^, potentially caused large slip in the near-trench area and promoted propagation of the 2011 rupture over a wide region^10^.

To understand the mechanism of such huge slip near the trench, the Japan Trench Fast Drilling Project (JFAST), conducted during Integrated Ocean Drilling Program (IODP) Expeditions 343/343T, penetrated the plate-boundary fault in the area that underwent large shallow slip during the Tohoku-oki earthquake (Fig. 1a). Highly sheared clay samples were successfully recovered from the plate boundary at 820 m depth below the seafloor^11^ (Fig. 1b). Great tsunamigenic earthquakes, such as the 1944 Tonankai (M_w_ 7.9), 1854 Ansei (M_w_ 8.4), and 1707 Hoei (M_w_ 8.6) earthquakes, have also occurred offshore of south-west Japan along the Nankai Trough, where the Philippine Sea plate is subducting beneath the Eurasian plate, and the predictions of the potential size of near-trench slip and triggered tsunami at the impending earthquake is now an urgent issue for the countries around the Pacific Rim. The Nankai Trough Seismogenic Zone Experiment (NanTroSEIZE), conducted during IODP Expeditions 314–316, targeted the rupture area of the 1944 Tonankai earthquake (Fig. 1c). Fault-rock samples were retrieved from the décollement (the shallow region of the plate-boundary fault) at 438 m depth below the seafloor in the Japan Trench, and from a megasplay fault (at 271 m depth) that branches from the plate interface in the Nankai Trough; ruptures in these regions are considered to have caused historical tsunamis^12^. Retrieved materials from both faults were characterized by a shear-localized dark fault gouge (Fig. 1c, arrowheads in the photographs), which were accompanied by thermal anomalies approximately 10 mm in width that were produced by a seismic rupture that propagated from the deeper plate interface^13^.

However, the varied lithological compositions of materials retrieved from actual fault zones at subduction boundaries, clayey in the Japan Trench^11^,^14^ and sandy in the Nankai Trough^15^, obscure the relationships between composition and fault weakening effects and slip potential, although large slip is generally considered to be facilitated by fault weakening due to thermally pressurized interstitial fluids and low shear resistance of impermeable, clayey fault materials^14^. The TP effect on fault slip may depend on the composition and properties of the fault rock and the condition of fluid pressure within the plate interface, but these dependencies have not been yet assessed quantitatively by using actual data. Thus, we focus here on the composition and properties of materials from two world-representative subduction faults, the Japan Trench and the Nankai Trough, to understand the potential for very large slip on these faults and, thus, extreme tsunamis.

We first acquire a quantitative data set of the mineral assemblages, dehydration weight loss amounts, fluid transport, and frictional properties of each fault materials retrieved from the plate-boundary fault in the Japan Trench and the megasplay fault in the Nankai Trough by using X-ray diffraction (XRD), simultaneous thermogravimetry–differential scanning calorimetry (TG-DSC), a high-pressure hydraulic apparatus, and a rotary shear apparatus (Methods). We then evaluate the potential for slip on these faults to cause an extreme tsunami hazard quantitatively by integrating these properties of actual fault materials and simulations of fault weakening and earthquake rupture propagation.

## Results

### Properties of the fault materials

**Figure 1:**
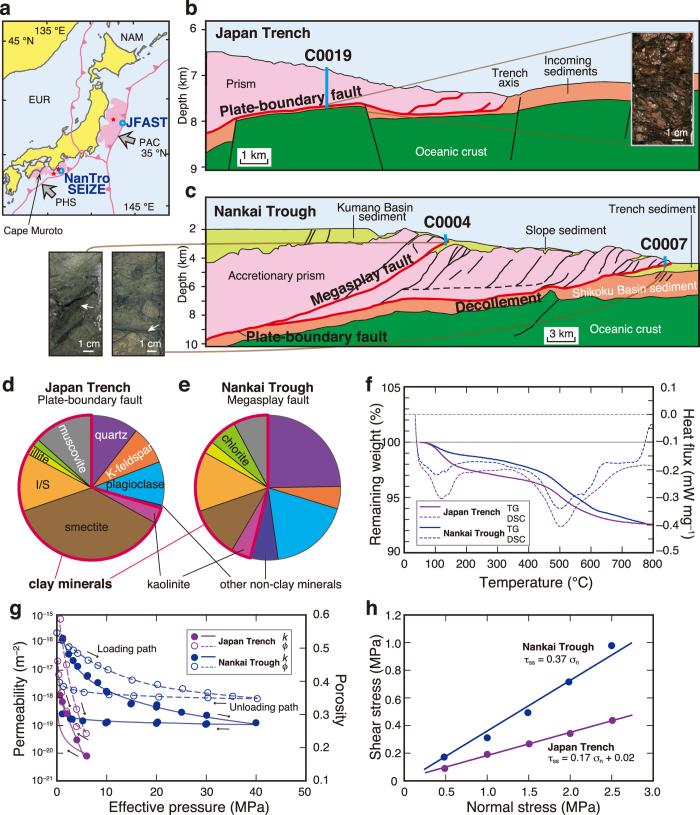
Drilling into rupture areas of the 1944 Tonankai and the 2011 Tohoku-oki earthquakes. (**a**) Tectonic setting of the Japanese islands and drilling locations of IODP Expeditions 314–316 (NanTroSEIZE) and 343/343T (JFAST). EUR, Eurasian plate; NAM, North American plate; PHS, Philippine Sea plate; PAC, Pacific plate; pink, rupture area; red star, hypocentre; open blue circles, drilling sites. (**b**) Section through drilling site C0019 based on seismic data around the Japan Trench^11^, and a photograph of the plate-boundary fault material. (**c**) Section based on seismic data around the Nankai Trough through drilling sites C0004 and C0007^15^ and photographs of the megasplay (left) and décollement (right) fault materials. (**d,e**) Mineral assemblages of the fault materials (**d**) plate-boundary fault of the Japan Trench; **e**, megasplay fault of the Nankai Trough). I/S, illite/smectite mixed-layer mineral (illite content >70%). The heavy red lines show the proportions of total clay minerals. (**f**) TG and DSC curves showing the relationships of total weight loss and heat flux to temperature. (**g**) Permeability (*k*) and porosity (*ϕ*) as a function of effective pressure. (**h**) Friction coefficients at a slip velocity of 0.01 m s^−1^.

The clay mineral content of the scaly clay from the Japan Trench was high (70 wt.%) and was dominated by smectite, whereas the dark gouge of the megasplay fault in the Nankai Trough was composed mainly of quartz and feldspar with relatively small amounts of clay minerals (46 wt.%) and calcite (Fig. 1d,e). During heating of the fault samples to 800 °C for the TG-DSC analyses, we detected a two-step endothermic weight loss in both samples that was caused by dehydration of interlayer water and dehydroxylation of hydrous minerals such as clay minerals^16^, respectively (Fig. 1f). Both the permeability and porosity decreased with increasing effective pressure (confining pressure minus interstitial-fluid pressure), and the scaly clay from the Japan Trench was relatively impermeable (Fig. 1g). In both samples, the dependence of shear stress on normal stress showed high linearity, and we determined the friction coefficient of the scaly clay from the Japan Trench to be 0.17 and that of the dark gouge from the megasplay fault in the Nankai Trough to be 0.37 (Fig. 1h). No sample from the décollement dark gouge in the Nankai Trough was available, but the properties of the fault material of the décollement resemble those of the megasplay fault (see Methods).

### Modelling of TP weakening

To quantify the TP effect on a subduction earthquake, we numerically simulated earthquake slip at shallow depths (1–10 km) by simultaneously solving the equations for one-dimensional heat and fluid diffusion, energy balance, and chemical kinetics. The pressurization of interstitial fluid (*P*_*f*_) at distance *x* from the centre of the slip zone in a direction normal to the fault plane is expressed as


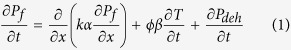


where *k* is permeability, *ϕ* is porosity, and *T* is temperature (for the definition of *α* and *β*, see Methods). Three factors contribute to pressurization during earthquake slip (corresponding in order to the three terms on the right side of the equation): fluid diffusion (hereafter referred to as the diffusion term), thermal expansion of interstitial fluid (thermal term), and fluid pressure generated by dehydration reactions (*P*_*deh*_, dehydration term)^16^. The fault zone temperature increases with slip (frictional heat = shear stress × slip distance × specific heat capacity^−1^ × density^−1^), but the increase is tempered as a result of the heat diffusion and the endothermic dehydration reaction^16^. In our simulation we applied realistic physical properties obtained from analyses and experiments using the fault-rock materials from the Japan Trench and the Nankai Trough. The *in situ P*_*f*_ value before an earthquake also affects dynamic slip^7^; a hydrostatic *P*_*f*_ was estimated in the Japan Trench^17^ but an overpressured *P*_*f*_ has been observed in the Nankai Trough^18^. We adopted hydrostatic *P*_*f*_ values for both in our simulations testing the material properties; however, for the Nankai Trough only, we also performed simulations using an overpressured *P*_*f*_ value. We assumed a 10-mm-thick slip zone (the thickness of the dark gouge in the Nankai Trough^13^) and computed temporal changes of interstitial fluid pressure, shear stress, and temperature by solving fluid and temperature diffusion equations coupled to dehydration kinetics by the finite-difference method (Methods).

**Figure 2:**
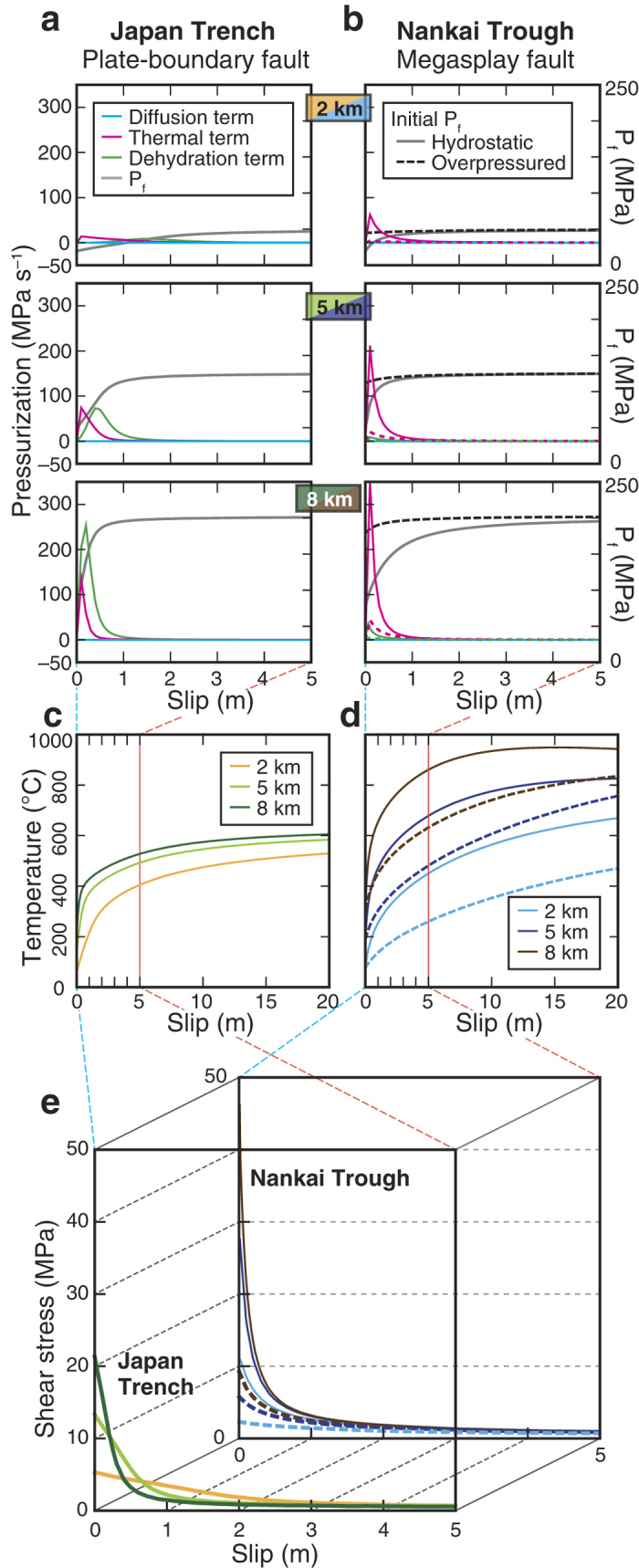
Dynamic fault weakening by thermal pressurization. (**a,b**) Contributions of the diffusion, thermal, and dehydration terms in equation (1) to the pressurization of interstitial fluid (*P*_*f*_) at the centre of the slip zone at representative depths of 2, 5 and 8 km below the seafloor. The right vertical axis corresponds to change of *P*_*f*_, including the three contributions, with slip. (**c,d**) Elevation of temperature with slip at the centre of the slip zone for the plate-boundary fault of the Japan Trench (left) and for the megasplay fault of the Nankai Trough (right). (**e**) Changes of shear stress averaged within the slip zone. The dashed curves show the results of simulations using the overpressured initial *P*_*f*_ condition in the Nankai Trough.

The results showed that for both the plate-boundary fault of the Japan Trench and the megasplay fault of the Nankai Trough, pressurization of the interstitial fluid started immediately after the beginning of slip and, after a rapid increase, reached an almost constant level (Fig. 2a,b). The contribution of the dehydration term at depths of 5 and 8 km was greater for the clayey Japan Trench material than for the sandy Nankai Trough material, but the thermal term had a greater effect on the Nankai Trough material. The effect of the diffusion term was very small for both fault materials. Simulated temperatures were higher for the Nankai Trough than for the Japan Trench (Fig. 2c,d). The larger amount of clay minerals in the Japan Trench fault rock led not only to a larger dehydration term but also to a lower friction coefficient and a smaller amount of interstitial fluid (low porosity, linearly affecting *P*_*f*_). In contrast, the small amount of clay minerals in the Nankai Trough material resulted in higher friction during slip and larger amounts of interstitial fluid (high porosity), leading to greater frictional heating and subsequent thermal expansion–pressurization of the interstitial fluid (thermal term). Clay mineral content, friction, and porosity have been shown experimentally to be correlated^19^, and the intensity of the TP effect is essentially determined by the complex interrelationships among these parameters. Although an abundance of clay minerals combined with low permeability and low friction^19^,^20^,^21^ has been proposed to be a key ingredient for producing large slip^11^,^14^, the sandy Nankai Trough material has a strong potential of more drastic weakening as a result of a more intense TP effect, compared with the clayey Japan Trench material (Fig. 2e). The high permeability (about 10^–16^ m^2^) of the sandy fault rock does not efficiently promote depressurization in the slip zone by fluid diffusion over the short duration of slip (Fig. 2b). From the viewpoint of the material properties, the effects of TP are stronger in the case of the Nankai fault. However, when the effects of an overpressured initial *P*_*f*_ are considered (that is, low effective normal stress on the fault before an earthquake), the levels of fluid pressurization and weakening become much smaller than in the case of a hydrostatic initial *P*_*f*_ (Fig. 2b,e). Therefore, the net effect of the TP weakening is smaller in the Nankai Trough than in the Japan Trench.

### Dynamic rupture simulations of near-trench slip

**Figure 3:**
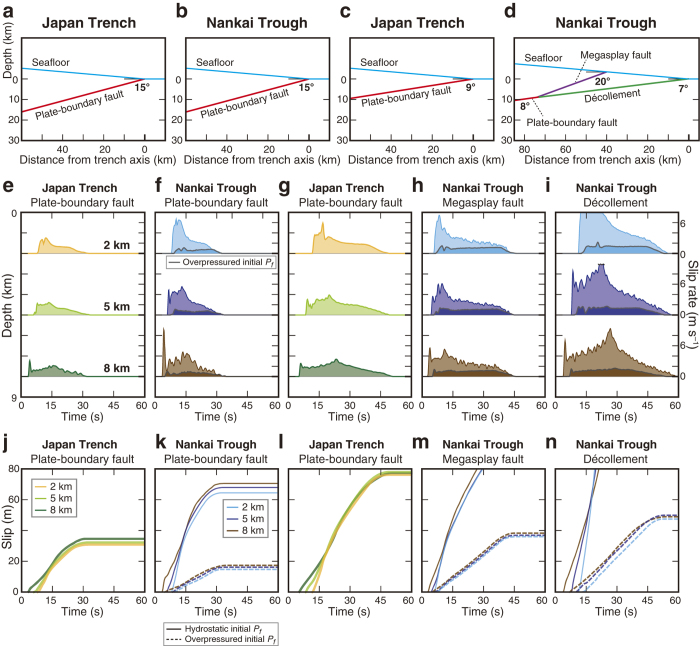
Spatio-temporal distribution of near-trench earthquake slip. (**a–d**) Fault geometries used in our dynamic rupture modelling: (**a**) ideal plate-boundary fault with 15° dip in the Japan Trench; (**b**) ideal plate-boundary fault with 15° dip in the Nankai Trough; (**c)** plate-boundary fault in the Japan Trench; (**d**) realistic branching geometry in the Nankai Trough. (**e–i**) Slip rate as a function of time at representative depths (2, 5 and 8 km) along the faults. (**j–n**) Accumulation of slip with time at each depth. The dashed curves show the results of simulations using the overpressured initial *P*_*f*_ condition.

To understand near-trench earthquake slip, we simulated two-dimensional dynamic rupture propagation using a slip-weakening friction law. The stress-versus-slip curves in our TP modelling were derived from the experimental data (Fig. 2e) using the actual fault materials from the Japan Trench and the Nankai Trough. To focus on the effects due to the differences in the fault-rock properties, we first used the same fault geometry for both simulations: a fault dipping at an angle of 15° embedded in a two-dimensional elastic half-space (Fig. 3a,b). Subsequently, we adopted more realistic fault geometries for the Japan Trench and the Nankai Trough (Fig. 3c,d), and for the Nankai Trough, we also performed simulations using an overpressured initial *P*_*f*_ condition. By using the spectral element method^22^, a nucleation procedure was prescribed and the results of the computation showed the direction and speed of the rupture propagation, which are primarily governed by a fault-weakening friction law constrained by the TP effect. Along the shallow part of the fault (0–10 km depth), fault-weakening properties such as yield shear stress and dynamic frictional stress determined by our fault-rock analyses were applied (Methods). We assumed the same friction law for the megasplay fault and the décollement in the Nankai Trough.

After nucleation at 11 km depth along the plate-boundary fault, the rupture propagated updip with a slip rate of approximately 1–3 and 2–6 m s^–1^ in the Japan Trench and the Nankai Trough, respectively (Fig. 3e,f), and for large distances of approximately 30 and 65 m, respectively (Fig. 3j,k). These results show that the properties of the sandy Nankai Trough material have the potential to allow a larger slip than those of the clayey Japan Trench material. Simulations using the realistic geometries reproduced a slip rate of approximately 2–6 m s^–1^ and a slip distance of approximately 75 m in the Japan Trench, and a slip rate of approximately 3–10 m s^–1^ and over 80 m of slip on the megasplay fault and décollement in the Nankai Trough (Fig. 3g–i,l–n). However, in simulations adopting an overpressured initial *P*_*f*_ in the Nankai Trough the slip was restrained to approximately 15 m along the 15°-dip interface, 35 m along the megasplay fault and 50 m along the décollement (Fig. 3k,m,n).

## Discussion

The large slip of about 75 m near the Japan Trench and the large stress drop reproduced by our simulations are well consistent with characteristics of the 2011 Tohoku-oki earthquake obtained by observations and surveys (50–80 m slip)^3^,^4^,^23^,^24^. Although drastic fault weakening can potentially occur in the sandy Nankai Trough material owing to a stronger TP effect than in the clayey Japan Trench material, the initial excess fluid pressure in the Nankai Trough^18^ crucially inhibits such weakening. Nevertheless, at least over 30 m slip was quantitatively predicted for both the megasplay fault and the décollement in the Nankai Trough, and this slip is consistent with historical records of large tsunamis accompanying past earthquakes^25^,^26^.

Our new approach to the assessment of extreme near-trench tsunamigenic slip based on the analysis of fault-rock materials is applicable to other plate-boundary faults (e.g., Barbados^27^ and Sumatra^28^), although future studies should also consider possible lateral variation in the lithological composition of the subduction fault materials: for example, rocks from the fault offshore of Cape Muroto along the Nankai Trough, approximately 200 km west of the drilling site of IODP Expeditions 314–316, have a higher clay mineral content (ca. 60 wt.%)^29^, probably owing to a difference in the clay mineral provenance^30^. The geological and physical properties in a fault zone should be sampled by drilling into subduction faults at multiple trench-parallel sites. Not only the properties of the fault material and the distribution of excess fluid pressure, but also the complexity of the rough plate–interface geometry and the slip-deficit rate at the interface might affect the rupture propagation^31^. Other fault weakening mechanisms, such as melting or other types of lubrication, might be triggered depending on the fault roughness, rock composition and interstitial-fluid distribution^32^. An integrated program of drilling into subduction faults, dense geophysical and seismological observations, and TP and dynamic rupture simulations such as those described here allow constraining earthquake source parameters by considering more realistic physical properties and rheologies of the fault materials typical of megathrust environments. This in turn should enable a more complete quantitative assessment of tsunamigenic potential and can contribute to a better understanding of megaquake physics.

## Methods

### Fault-rock samples

Although there are splay faults and a plate-boundary fault in both the Japan Trench^2^ and the Nankai Trough^12^, fault-rock samples from actual tsunamigenic faults were retrieved only from the plate-boundary fault in the Japan Trench^11^ and the megasplay fault in the Nankai Trough^13^. In the Nankai Trough, the megasplay fault was shown by tsunami waveform inversion analyses to have slipped during the 1944 Tonankai earthquake^12^,^33^, but the dynamic constraints on the upgoing rupture, that is, whether it branched out to the megasplay fault or continued propagating along the décollement, was examined by a three-dimensional finite element method. The result showed that if a barrier such as a seamount existed along the strike of the plate-boundary fault, the rupture would propagate to the megasplay fault, but if the stress distribution was homogeneous, the rupture would propagate along the décollement^34^. However, because it cannot be predicted whether the upgoing rupture of the next large earthquake will branch out to the megasplay fault or continue propagating along the décollement, in this study we considered both cases. Because no sample from the décollement dark gouge in the Nankai Trough was available, we referred to previously published data to constrain its properties^35^,^36^,^37^.

### XRD analysis

XRD spectra of fault-rock samples retrieved from the plate-boundary fault in the Japan Trench and the megasplay fault in the Nankai Trough were obtained with a Spectris PANalytical X’Pert PRO MPD spectrometer with monochromatized Cu Kα radiation operated at 45 kV, 40 mA, using a step width of 0.004° (∆2θ), 0.25° divergence and anti-scattering slits, and a high-speed semiconductor array detector. In addition, the samples were blended with α-alumina (25 wt.%) as an internal standard and mounted on XRD glass holders by the side-load method to minimize preferred alignment of the phyllosilicates. The mineral assemblages were determined by using the RockJock program^38^, which fits stored integrated XRD patterns of standard minerals to the XRD patterns of the samples to determine the weight percent values of component minerals in the samples. Representative XRD patterns and an original result sheet are shown in Supplementary Figure S1 and Supplementary Table S1, respectively. Data of mineral assemblages in the plate-boundary fault material from the Japan Trench obtained by our XRD-RockJock analyses agreed well with those for similar materials retrieved from this fault^39^,^40^,^41^.

Because no sample from the décollement dark gouge in the Nankai Trough was available, we examined the ratio of the total clay mineral content of the megasplay fault material to that in fault rocks from the décollement, determined from actual samples^36^. The ratio, 1.058, indicates that the clay mineral contents of materials from the two faults were similar. Grain densities of the megasplay fault and décollement were 2.66 and 2.68 g cm^–1^, respectively^36^, which supports the inferred similarity of their mineral compositions.

### TG–DSC analysis

TG-DSC was carried out with a Netzsch STA 449 C Jupiter balance; the resolutions of TG, DSC, and temperature were 1 μg, 1.25 μW, and 0.01 °C, respectively. Approximately 30 mg of sample was placed in a covered Pt_90_Rh_10_ crucible and heated from room temperature to 800 °C at a rate of 5–20 °C min^–1^ under a flow of nitrogen gas (50 mL min^–1^). Using the kinetic software Netzsch Thermokinetics with constraint by Friedman analysis^42^, the most appropriate kinetic function and parameters such as activation energy were assessed by statistical analysis (*F*-test) (Supplementary Table S2). Details of this sequential procedure are described in ref. 16.

### Fluid transport property measurement

Because no laboratory hydraulic data were obtained from the actual narrow dark gouge within the megasplay fault at the Nankai Trough (arrowheads in photograph of Fig. 1c), permeability and porosity of the sample were measured by using a high-pressure apparatus at a confining pressure of 1–40 MPa and room temperature (20–25 °C); specific storage was calculated from the relationship between porosity and effective pressure, following the procedure described in ref. 39. Because of the small amount of dark gouge in the sample from 271.1 m depth below the seafloor of the Nankai Trough, another scaly clay sample collected at 271.4 m depth that had almost the same mineral assemblage as the dark gouge (Supplementary Table S1) was used for this procedure. The resulting data of permeability and porosity were fitted as power-law functions of effective pressures (Supplementary Table S2).

Data for the plate-boundary fault in the Japan Trench were obtained from ref. 37 (run number, CCT299). Our measured values of transport properties agreed well with previous data of similar fault materials from the megasplay fault (approximately 10^–19^ to 10^–17^ m^2^)^36^,^37^,^43^,^44^. Permeabilities of the megasplay fault and décollement materials in the Nankai Trough were previously reported to have similar values and to depend similarly on the confining stress^36^,^37^; therefore, we used the transport properties obtained from our measurements of the megasplay fault for the décollement as well.

### Friction experiment

The friction coefficient was measured by using a rotary-shear apparatus with slip-rate control^45^. The friction coefficient is generally velocity dependent and shows a peak value at a velocity of around 0.01 m s^–1^, independent of the rock type (e.g., ref. 46,47). In addition, values at high slip velocities of 0.1–1.0 m s^–1^ reflect the effects of weakening mechanisms such as powder lubrication^48^ and thermal decomposition^49^. We thus adopted a velocity of 0.01 m s^–1^ (equivalent slip velocity^45^) as representative of peak friction during coseismic slip. Approximately 500 mg of water-saturated powdered sample was placed between the ends of two cylinders of permeable sandstone and sealed in a polytetrafluoroethylene sleeve to prevent leaks. The shear stress was measured with a slip displacement of 1–3 m and normal stress of 0.5–2.5 MPa (Supplementary Figure S2) after correction for friction between the rock cylinders and the sleeve^45^.

In our experiments, the friction coefficient of the scaly clay from the Japan Trench was determined to be 0.17, and that of the dark gouge from the megasplay fault in the Nankai Trough was 0.37. Both values agree well with previous laboratory data obtained for slip rates from about 0.01 to 1 m s^–1^: approximately 0.17 at 1.3 m s^–1^ for similar fault material from the Japan Trench^14^ and 0.14 at 0.013 m s^–1^ for pelagic clay with a mineral assemblage similar to that of the Japan Trench^50^; 0.375 at 0.003 m s^–1^ for the megasplay fault material^35^ and approximately 0.34 at 1.3 m s^–1^ for the décollement material in the Nankai Trough^14^. Because no sample from the décollement dark gouge in the Nankai Trough was available, for our modelling of the décollement we adopted the friction coefficient value that we obtained for the megasplay fault dark gouge.

### Thermal pressurization modelling

Thermal pressurization (TP) is a significant fault weakening mechanism during earthquake slip^8^,^9^. Comprehensive theoretical works on TP have been published in the past two decades (e.g., refs 9,51, 52, 53, 54). Laboratory data on natural fault gouges indicate that TP might indeed be an effective fault weakening mechanism during real earthquakes^55^,^56^ and an experimental demonstration of TP was recently achieved by direct measurements of interstitial-fluid pressure and temperature during friction experiments at slip rates of the order of 1 m s^–1 ^57. In addition, coseismic mineral dehydration and decomposition and their reaction kinetics have been incorporated into the TP theoretical framework^16^,^58^,^59^,^60^,^61^. Remarkably, dehydration reactions act as a source of interstitial-fluid pressure, and also as a temperature sink because they are endothermic^16^,^59^,^60^,^63^. Dilatancy of fault zone materials, which acts as a fluid pressure sink, is an important competing factor that affects TP^9^,^64^,^65^,^66^. Both dilation and compaction of rocks during shear deformation involve complicated physico-chemical processes: loading-induced microcracking^67^,^68^,^69^,^70^; thermal stress cracking^71^,^72^; transformation of intergranular structure (rearrangement of grain packing) related to the consolidation state^73^,^74^,^75^; and chemically activated cracking caused by the replacement of stress-supporting atomic bonds close to crack tips with weaker bonds such as hydrogen bonds^76^,^77^. Dilatancy-induced suction reduces interstitial fluid pressure and increases effective normal stress, which impedes further crack growth, a well-known mechanism called dilatancy hardening^68^,^78^. Metre-scale dilatant fractures, observed by borehole electrical resistivity measurements^79^,^80^ and in field investigations of the San Andreas fault^81^, also act as a sink for interstitial fluid pressure. Microcrack-induced dilatancy has been incorporated into the TP theoretical framework^9^,^64^,^65^. However, according to a recent theoretical simulation study, dilatancy hardening is unlikely to overcome TP weakening at seismic slip rates^82^. In addition, under natural fault-rock conditions, the generation of dilation by microcracking and other processes is difficult to assess quantitatively, and there are currently no relevant data from the fault materials of the Japan Trench and the Nankai Trough. A shortening of axial displacement was observed during our friction experiments (Supplementary Figure S2), indicating that compaction due to rearrangement of grain packing occurred, but these data may be applicable only under our experimental conditions (0.01 m s^–1^ slip rate and low normal stress of <2.5 MPa). Therefore, we ignored dilatancy effects in our TP modelling, except for the volume change induced by the dehydration reaction described below.

We evaluated the TP dynamic fault-weakening effect by employing a one-dimensional heat and fluid diffusion model in which diffusion occurs normal to the fault plane^53^, and into which we incorporated fluid release and energy loss by dehydration. The temperature *T* at distance *x* from the centre of the slip zone is governed by the heat balance at time *t* as follows:


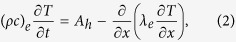


where (*ρ*c)_*e*_ is equivalent specific heat capacity, *ρ* is density, *c* is specific heat, *A*_*h*_ is heat production rate, and *λ*_*e*_ is equivalent thermal conductivity. The values of (*ρ*c)_*e*_ and *λ*_*e*_ were deduced from those of the solid matrix and the interstitial fluid, respectively^16^,^39^. Under the assumption that total frictional work is converted to heat and that the strain rate is constant within the slip zone, *A*_*h*_ is expressed as


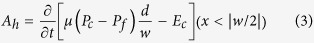



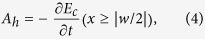


where *μ* is the friction coefficient, *P*_*c*_ is confining pressure, *d* is slip displacement, *w* is the thickness of the slip zone, and *E*_*c*_ is the energy taken up by endothermic dehydration. *E*_*c*_ is related to the chemical kinetics and the resultant reacted fraction, density of the reactant, and enthalpy of the reaction^16^. The fluid diffusion equation, expressed as equation (1), was deduced from the combination of the mass conservation law of the fluid phase for porous media and that of the solid phase (minerals), Darcy’s law, and Terzaghi’s effective pressure law, where α is 1/(specific storage × fluid viscosity), *β* is 1/(coefficient of thermal expansion of fluid – coefficient of thermal expansion of solid minerals) and *P*_*deh*_ is related to the amount of weight loss in the TG data (amount of water expelled) and the reacted fraction^16^. Nonlinearity of hydraulic properties was considered by using laboratory data on the fault-rock materials. A constant value of *μ*, determined by our friction experiment at 0.01 m s^–1^ slip velocity, was adopted as representative during coseismic slip, because values at high-slip velocities of 0.1–1.0 m s^–1^ reflect the complex effects of weakening mechanisms such as powder lubrication^48^ and thermal decomposition^49^, as described above. The thickness of the slip zone affects the resulting stress change^16^,^39^, but we assumed a thickness of 1 cm for both the Japan Trench and the Nankai Trough because our focus was on the effect of the different fault-rock properties. Initial temperature at each depth is assumed to be linearly dependent on depth calculated from the temperature at the seafloor and the geothermal gradient. A hydrostatic *P*_*f*_ was estimated for the Japan Trench^17^, but an overpressured *P*_*f*_ has been reported for the Nankai Trough: the normalized pore pressure ratio (=(pore-fluid pressure – hydrostatic pressure)/(confining pressure – hydrostatic pressure)) in and around the megasplay fault and the décollement has been determined to be approximately 0.8 by integrating high-resolution seismic velocity distribution and frequency-domain waveform tomography^18^. The overpressured *P*_*f*_ in the Nankai Trough has been considered to cause shallow slow earthquakes^83^,^84^,^85^,^86^. In our examination of the effect of the different fault-rock properties, we first assumed hydrostatic initial values of *P*_*f*_ at each depth for both the Japan Trench and the Nankai Trough. However, in subsequent simulations, for only the Nankai Trough, we also considered the case of an overpressured initial *P*_*f*_. We set the depth where TP occurs to be shallower than 10 km for both cases because deep materials of subduction faults might have different compositions and properties owing to complex mineral reactions (e.g., low-grade metamorphism^87^). All parameters for this modelling, determined by our experiments or referred from the previous reports, are summarized in Supplementary Table S2. By using the finite difference method (time increment of 0.001 s and grid size of 0.5 mm), these equations (1, 2, 3, 4), the dehydration kinetics, and the volume fraction rate by dehydration were simultaneously solved for a constant slip velocity of 1 m s^–1^, and the temporal evolution of temperature and of interstitial-fluid pressure distributions in the slip zone were obtained. The changes of shear stress corrected by averaging within the slip zone and by normalization to the initial value are shown in Supplementary Figure S3.

The simulations using a hydrostatic initial *P*_*f*_ for the plate boundary fault in the Japan Trench and the megasplay fault in the Nankai Trough showed that extreme weakening and low fracture energy (generally defined as the integral of the curve of strength-minus-residual-strength versus slip, also known as breakdown work^88^) resulted from an intense TP effect (Fig. 2e, Supplementary Figure S3). These features favour the propagation of a rupture^88^,^89^. In addition, wet clayey material during slip at a high rate (~1 m s^–1^) was suggested to be subject to TP, which led to small fracture energy and facilitated large rupture propagation^90^. For the quantitative evaluation of the magnitude of slip in the Japan Trench and the Nankai Trough, we performed dynamic rupture modelling as described in the next section.

### Dynamic earthquake rupture modelling

As in previous two-dimensional dynamic rupture simulations of the Tohoku-oki earthquake^5^,^91^, we used the spectral element method (SEM2DPACK)^22^ with a grid spacing of 1 km. A uniform elastic medium was assumed with a density of 2600 kg m^–3^, P-wave velocity of 6.30 km s^–1^, and S-wave velocity of 3.54 km s^–1^. For both the Japan Trench and the Nankai Trough modelling, we used the same material parameters for the medium to isolate the differences in dynamic rupture propagation due to the different fault-rock properties, the fault geometry, and the initial state of *P*_*f*_. For the simulations with realistic fault geometry, the dips were set to 9° for the plate boundary fault in the Japan Trench^92^ and to 8°, 20° and 7° for the deep portion of the plate-boundary fault, the megasplay fault and the décollement in the Nankai Trough, respectively^93^.

Rate- and state-dependent friction (RSF) laws have been developed to describe experimental rock friction results obtained at relatively low slip rates (e.g., ref 94, 95, 96, 97, 98, 99). Although the validity of these formulations has been clearly verified for relatively slow slip velocities in many experimental studies (e.g., refs 69,100, 101, 102), and they have been applied to explain fault behaviour during shallow, slow earthquakes along plate interfaces^86^,^103^, TP weakening can dominate RSF weakening during earthquake nucleation at slip speeds in excess of roughly 10^–4^ to 10^–2^ m s^–1 ^82. During dynamic rupture, such high slip rates are rapidly exceeded soon after the passage of the rupture front. Thus, RSF weakening is not dominant during the dynamic phase of seismic slip on crustal faults. In our model, we applied the exponential slip-weakening friction law^104^, which is consistent with the stress-versus-slip curve obtained by our TP modelling at high slip rate constrained with the experimental data (Fig. 2e):


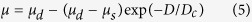


where *μ* is the friction coefficient as a function of slip, *μ*_d_ is the dynamic friction coefficient (friction coefficient during the stable sliding), *μ*_s_ is the static friction coefficient (friction coefficient at the yielding stress), *D* is the slip, and *D*_c_ is the critical slip distance (necessary slip to reach the stable sliding). A similar exponential slip-weakening behaviour is known to result during adiabatic, undrained TP at constant slip velocity^51^. The shear stress versus slip curves resulting from our TP modelling (Fig. 2e and Supplementary Figure S3) were fitted by the friction law (5) using a non-linear least squares method to obtain the values of *μ*_d_, *μ*_s_ and *D*_c_. We emphasize that the values of *μ*_d_ and *μ*_s_ involve the effect of interstitial-fluid pressurization. An example based on the plate-boundary fault in the Japan Trench at 1 km depth is shown in Supplementary Figure S4. The initial stress was set as the friction coefficient at the ultraslow slip rate of 10^–7^ m s^–1^. For the Japan Trench, a coefficient of 0.07 was obtained by extrapolating from laboratory observation at slip rates ranging from 10^–4^ to 10^–1^ m s^–1^^50^. For the Nankai Trough, a value of 0.154 was applied by assuming a similar stress ratio S (= strength excess/stress drop^7^,^105^,^106^; around 1.4) to the Japan Trench. Values of the S ratio affect the rupture speed and slip rate: higher S ratios result in slower rupture speeds and lower slip rates^105^,^107^. However, there are currently no relevant data from which to derive absolute values for the initial shear stress from either the Japan Trench or the Nankai Trough, so we applied similar S ratios for both. Rupture nucleation was achieved in our simulations by setting the yield strength slightly lower than the initial shear stress over a prescribed depth range, centred at a depth of 11 km and just large enough to trigger spontaneous rupture propagation without anomalously long acceleration stage^107^ (Supplementary Figure S5c,e).

Values of the shear strength and *D*_c_ used to specify the stress curve (fitted curve in Supplementary Figure S4) in the shallow part of the fault (1–10 km depth) are summarized in Supplementary Tables S3 and S4 under hydrostatic and overpressured initial *P*_*f*_ conditions, respectively, together with the correlation coefficients of the curve-fitting process. For the décollement in the Nankai Trough, we assumed the same friction law as for the megasplay fault. The middle depth part of the fault (11–20 km) was assumed to have a stress drop of 3 MPa, which is a representative value for interplate earthquakes^108^. In the deeper region below 21 km, a negative stress drop (dynamic frictional stress >initial shear stress) was assumed to prevent the rupture propagation into the deeper part. This study focuses on the slip behaviour near the trench and we wish to avoid the complications of the deeper portion of the fault by using simple assumptions that other parameters were constant below 11 km depth. Model parameters for our dynamic rupture simulation are shown for the whole region, including the area of slip strengthening, in Supplementary Figure S5.

The slip rate and accumulated slip as functions of time at representative depths (2, 5 and 8 km) along the faults were shown in Fig. 3 together with the model geometries, and here we show the more complete evolutions of slip as a function of time and depth on the fault (Supplementary Figures S6 and S7). Features of the time evolution of slip described in the main text (Fig. 3j–n) can be seen more clearly in these figures.

Dip of the fault affects the amount of slip because of the difference of the updip rupture length. For the Japan Trench, the dip of 9°, compared to 15°, causes larger slip because of the longer distance to the trench (Fig. 3j,l). For the Nankai megasplay fault model, compared to the simple model with constant 15° dip, the longer rupture length between the initiation point (11 km depth) and the branching point (9 km depth) along the deep plate-interface with 8° dip produces larger slip even though the rupture subsequently propagates along the more steeply 20°-dip megasplay fault (Fig. 3k,m).

The resulting frequency spectra of slip rate are shown in Supplementary Figure S8. For all cases, the high-frequency content of slip (0.1–0.5 Hz) at all depths was relatively low compared with the low-frequency content (<0.1 Hz). The stress ratio S controlling the rupture velocity^105^ was similar for both cases, but the Nankai Trough had a shorter critical slip distance than the Japan Trench in the shallow region under hydrostatic initial *P*_*f*_ conditions.

Because value of the S ratio is reported to affect the rupture speed and slip rate^105^,^107^, we have tested how the value affects the characteristics of rupture propagation by running new simulations with different initial shear stresses, corresponding to different S ratios (0.8 and 2.0), on the upper 10 km for the model with 15 degrees dip. The resultant data were shown in Supplementary Figure S9: higher S ratios result in lower slip rates and lower accumulated slips while lower S ratios show the opposite trends. Although the absolute values of slip change, the relative differences between the Japan Trench and Nankai Trough remain the same. We confirm that for different S ratios, the Japan Trench has potential large slip, and the Nankai Trough shows much larger slip for the hydrostatic case but more restrained slip, which is consistent with historical records^25^,^26^, for the overpressured case.

We here discuss other uncertainties in our modelling. The simulated slip rates for both cases (2 to 10 m s^–1^) were higher than the assumed constant value of 1 m s^–1^ used for the TP modelling and 0.01 m s^–1^ under the rotary-shear experiments. A higher slip rate (3 m s^–1^) causes faster pressurization by the dehydration and thermal terms (equation 1) for the Japan Trench and by the thermal term for the Nankai Trough, leading to more intense fault weakening, together with shorter *D*_c_ (Supplementary Figure S10a–d). As a result, more seismic energy could be radiated. However, some uncertainty in the quantitative values of our results arises from an inconsistency between the slip velocities assumed in our TP modelling and obtained from our dynamic rupture simulation modelling. In addition, we assessed the effect of the thickness of the slip zone on the weakening, because a slip zone of an ancient subduction fault that formed at 2.5–5.5 km depth and is now exposed adjacent to the Kure Melange in the Shimanto accretionary complex is 5–10 mm thick^109^. The slip zone is characterized by marked geochemical anomalies in the concentrations of fluid-mobile trace elements (Sr, Cs, Rb and Li) and in the Sr isotope ratio, indicating that a high-temperature fluid (>350 °C) was present during the earthquake and thus that TP occurred^110^. A thinner slip zone, 5 mm, also causes faster pressurization and results in shorter *D*_c_ and smaller dynamic frictional stress for both the Japan Trench and the Nankai Trough (Supplementary Figure S10e–h). However, in all results with a 3 m s^–1^ slip rate or a 5 mm slip-zone thickness, the Nankai Trough always showed more drastic weakening than the Japan Trench, indicating that the fault-rock properties of the Nankai Trough have more potential for producing a larger slip than those of the Japan Trench under the same initial *P*_*f*_ conditions. An integrated numerical analysis with TP modelling incorporated in the dynamic rupture simulation, based on laboratory-constrained fault zone rock properties, is required to improve the quantification of the slip potential near the trench along subduction faults.

## Additional Information

**How to cite this article**: Hirono, T. *et al*. Near-trench slip potential of megaquakes evaluated from fault properties and conditions. *Sci. Rep.* **6**, 28184; doi: 10.1038/srep28184 (2016).

## Supplementary Material

Supplementary Information
