# Supplementary material for: Near-trench slip potential of megaquakes evaluated from fault properties and conditions
Source: Sci Rep. 2016 Jun 20;6:28184. doi: 10.1038/srep28184 (PMC4913312; doi:10.1038/srep28184)
Supplement: Supplementary Information [file srep28184-s1.pdf]

SUPPLEMENTARY INFORMATION

Near-trench slip potential of megaquakes evaluated from fault properties and conditions

Tetsuro Hirono[1*], Kenichi Tsuda[2], Wataru Tanikawa[3], Jean-Paul Ampuero[4], Bunichiro Shibazaki[5], Masataka Kinoshita[6] and James J. Mori[7]

[1] Department of Earth and Space Science, Graduate School of Science, Osaka University, Toyonaka, Osaka 560-0043, Japan.

[2] Center for Safety and Reliability Engineering, Institute of Technology Shimizu Corporation, Koto, Tokyo 135-8530, Japan.

[3] Kochi Institute for Core Sample Research, Japan Agency for Marine–Earth Science and Technology, Nankoku, Kochi 783-8502, Japan.

[4] Division of Geological and Planetary Sciences, California Institute of Technology, Pasadena, CA 91125, U.S.A.

[5] International Institute of Seismology and Earthquake Engineering, Building Research Institute, Tsukuba, Ibaraki 305-0802, Japan.

[6] Earthquake Research Institute, University of Tokyo, Bunkyo, Tokyo 113-0032, Japan.

[7] Earthquake Hazards Division, Disaster Prevention Research Institute, Kyoto University, Uji, Kyoto 611-0011, Japan.

* Corresponding author. Contact: hirono@ess.sci.osaka-u.ac.jp

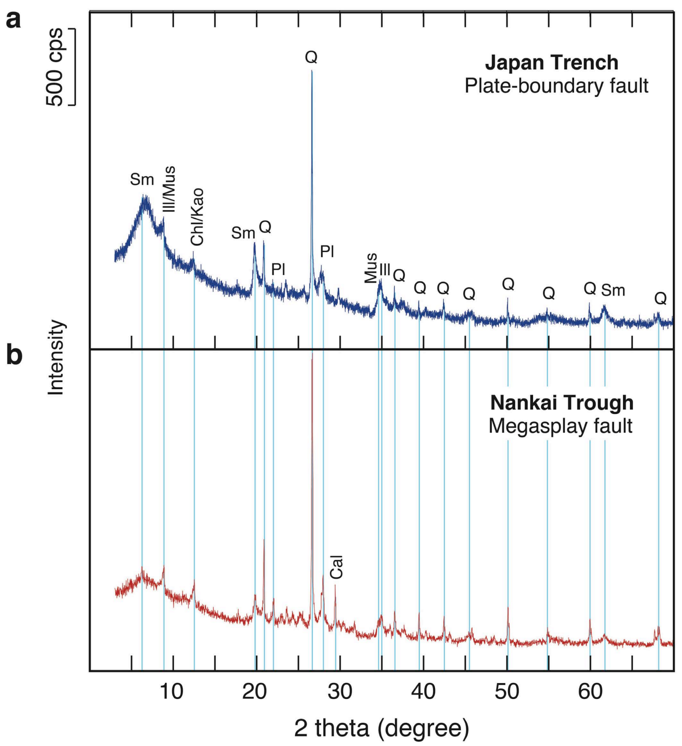

Supplementary Figure S1. XRD patterns of the fault-zone materials. a, Plate-boundary fault of the Japan Trench. **b,** Megasplay fault of the Nankai Trough. Sm, smectite; Ill, illite; Mus, muscovite; Chl, chlorite; Kao, kaolinite; Q, quartz; Pl, plagioclase; Cal, calcite; cps, counts per second.

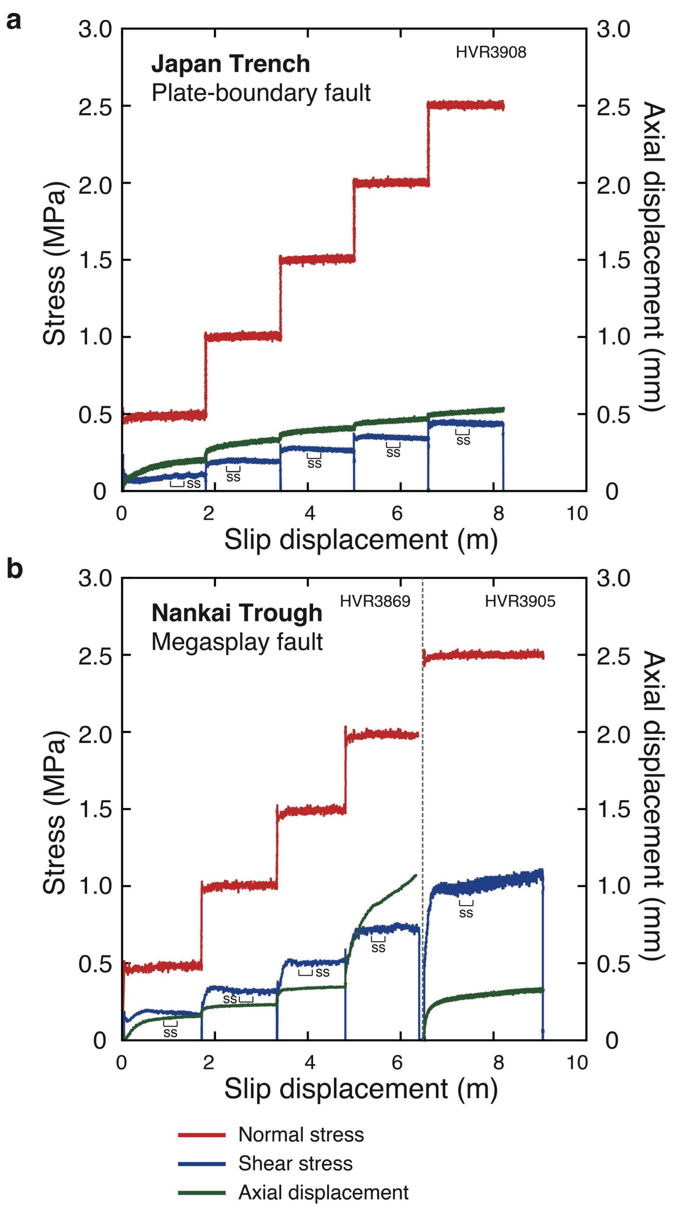

Supplementary Figure S2. Frictional behaviours of the fault-zone materials. a, Plate-boundary fault of the Japan Trench. **b,** Megasplay fault of the Nankai Trough. Experimental parameters were set to an equivalent slip velocity[45] of 0.01 m s^{-1}, normal stresses of 0.5–2.5 MPa, and slip displacements of 1–3 m. The steady state (ss) of the friction was confirmed at each normal stress to determine the friction coefficient. At the end of run HVR3869 sample leakage occurred, so an additional run (HVR3905) was performed.

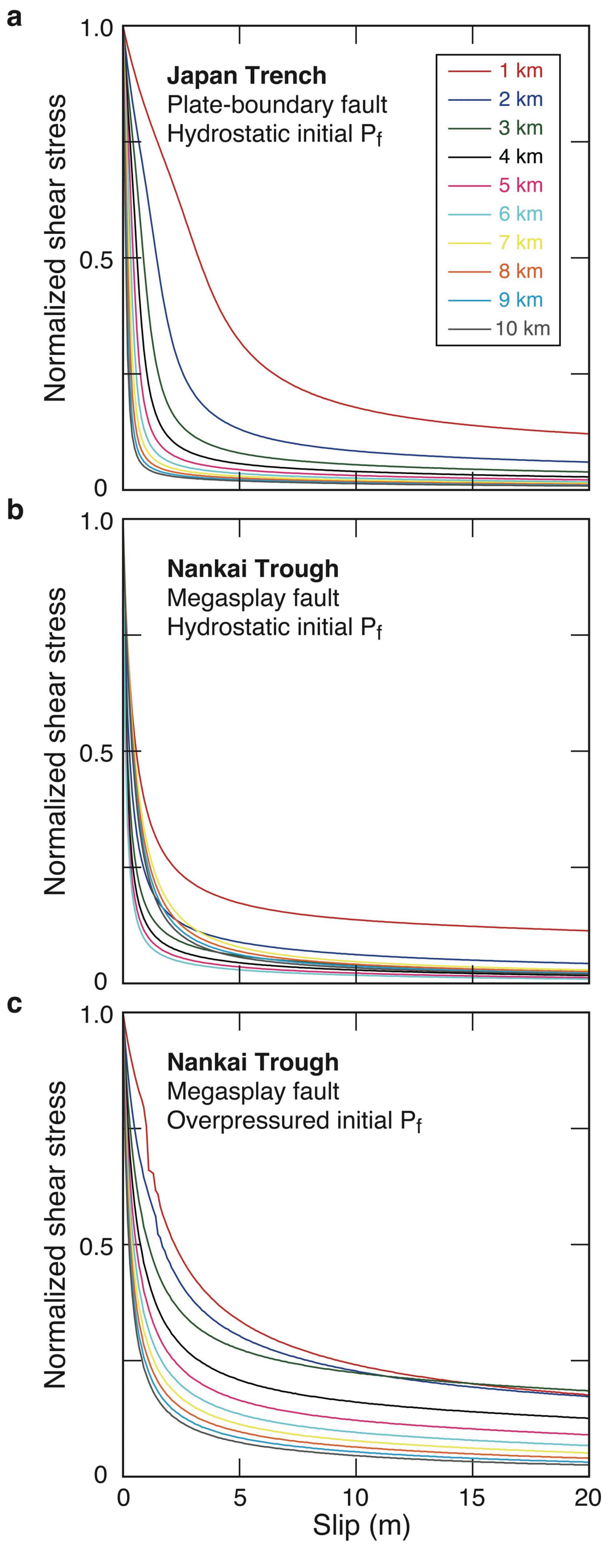

Supplementary Figure S3. Changes of shear stress normalized by the initial value with slip at all depths. a, Plate-boundary fault of the Japan Trench. **b,** Megasplay fault of the Nankai Trough under hydrostatic initial P_f condition. **c,** Megasplay fault of the Nankai Trough under overpressured initial P_f condition.

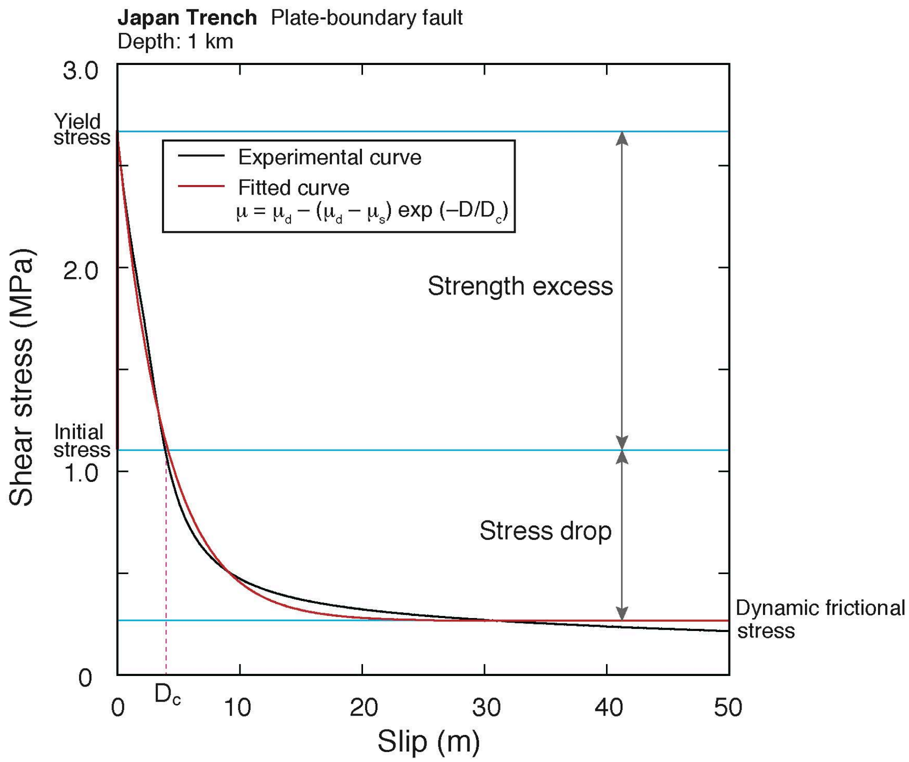

Supplementary Figure S4. Curve fitting for dynamic rupture modelling. An example from the Japan Trench plate-boundary fault at 1 km depth (dip, 15°). Strength excess = yield shear stress – initial shear stress, and stress drop = initial shear stress – dynamic frictional stress. D_c, Critical slip distance.

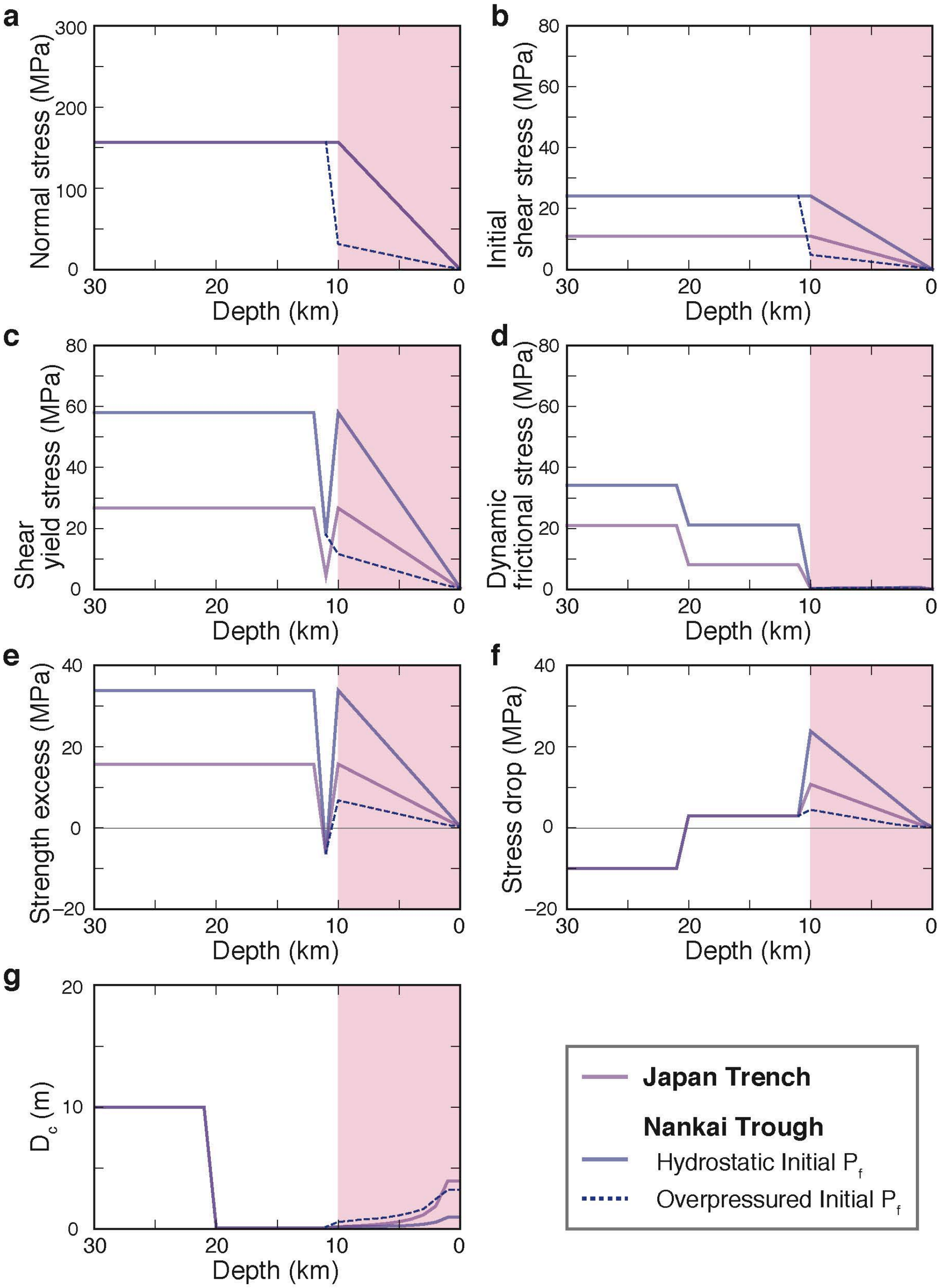

Supplementary Figure S5. Model parameters for dynamic rupture propagation. a, Normal stress. **b,** Initial shear stress. **c,** Yield shear stress. **d,** Dynamic frictional stress. **e,** Strength excess. **f,** Stress drop. **g,** Critical slip distance (D_c). Earthquake nucleation was started at 11 km depth by prescribing a local reduction of yield stress (**c** and **e**). Pink shading shows the region where thermal pressurization occurred in our modelling. The dashed curves show the parameters for the simulations using the overpressured initial P_f condition at the Nankai Trough. Depth does not correspond to the distance from the trench along dip.

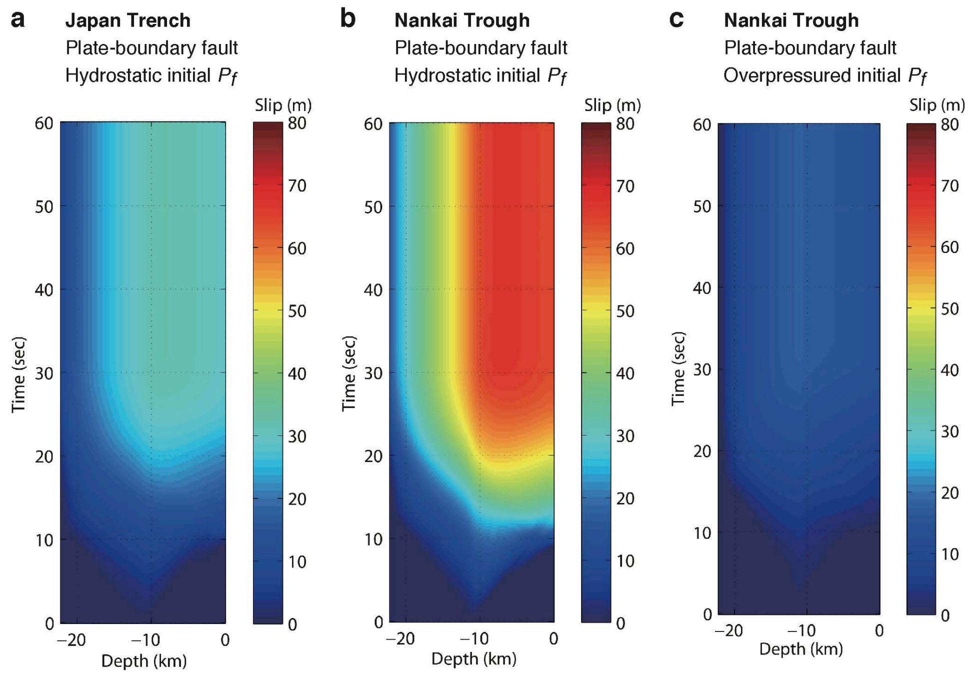

Supplementary Figure S6. Time evolution of slip as a function of depth. a, Plate-boundary fault (dip 15˚) in the Japan Trench at hydrostatic initial P_f condition. **b,** Plate-boundary fault (dip 15˚) in the Nankai Trough at hydrostatic initial P_f condition. **c,** Plate-boundary fault (dip 15˚) in the Nankai Trough at overpressured initial P_f condition. Depth does not correspond to the distance from the trench along dip.

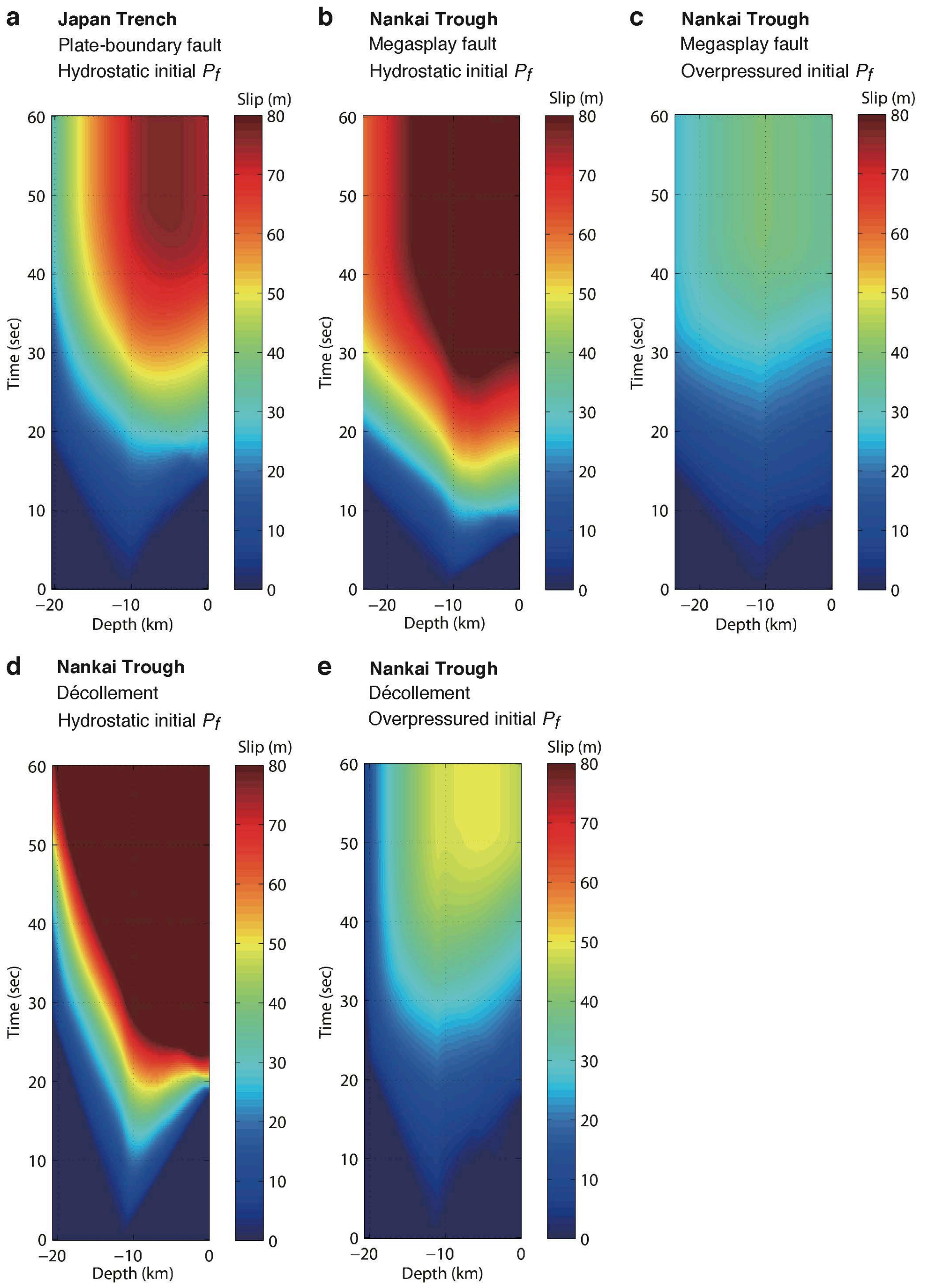

Supplementary Figure S7. Time evolution of slip as a function of depth. a, Plate-boundary fault (dip 9°) in the Japan Trench at hydrostatic initial P_f condition. **b,** Megasplay fault (dip 20°) in the Nankai Trough at hydrostatic initial P_f condition. **c,** Megasplay fault (dip 20°) in the Nankai Trough at overpressured initial P_f condition. **d,** Décollement (dip 7°) at the Nankai Trough at hydrostatic initial P_f condition. **e,** Décollement (dip 7°) at the Nankai Trough at hydrostatic initial P_f condition.

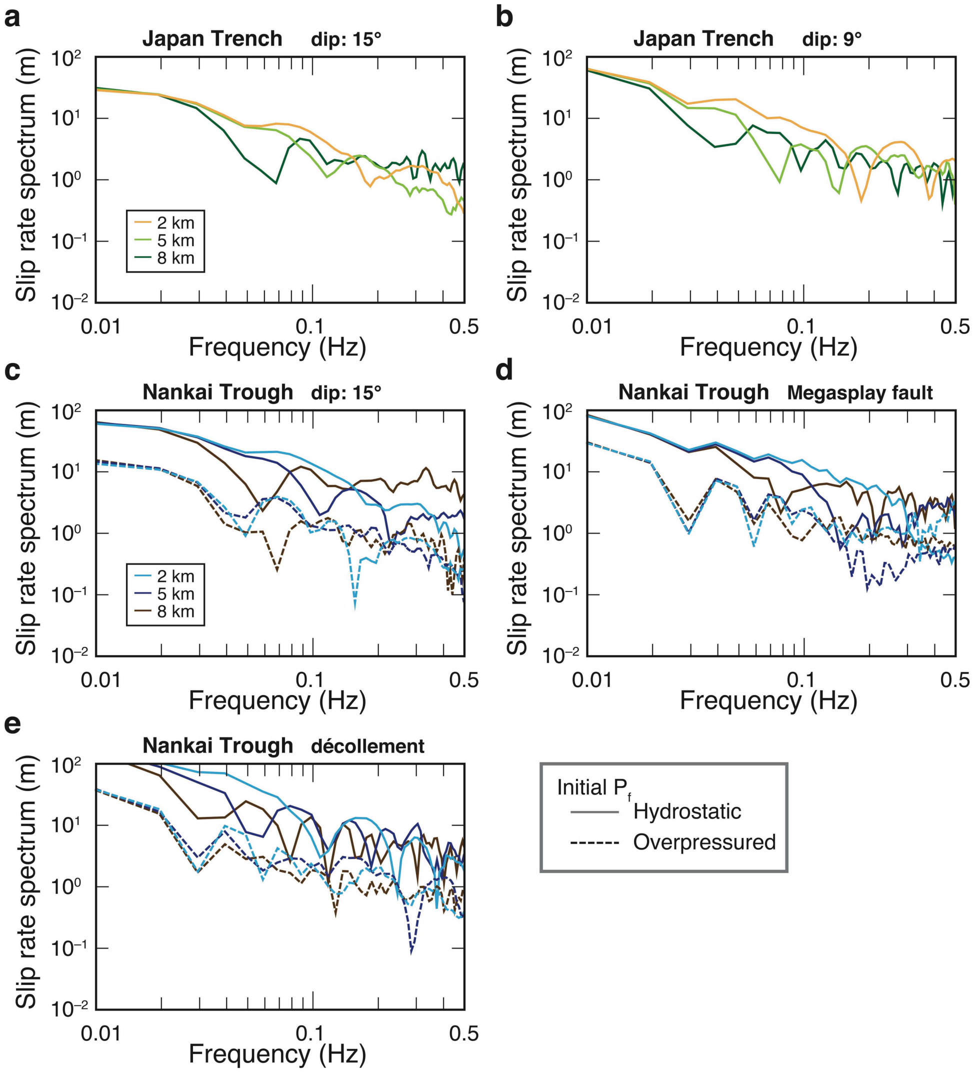

Supplementary Figure S8. Frequency spectra of slip rate. a, Ideal plate interface with 15° dip in the Japan Trench. **b,** Plate-boundary fault in the Japan Trench. **c,** Ideal plate interface with 15° dip in the Nankai Trough. **d,** Megasplay fault at the Nankai Trough. **e,** Décollement at the Nankai Trough. The dashed curves show the results of simulations using the overpressured initial P_f condition.

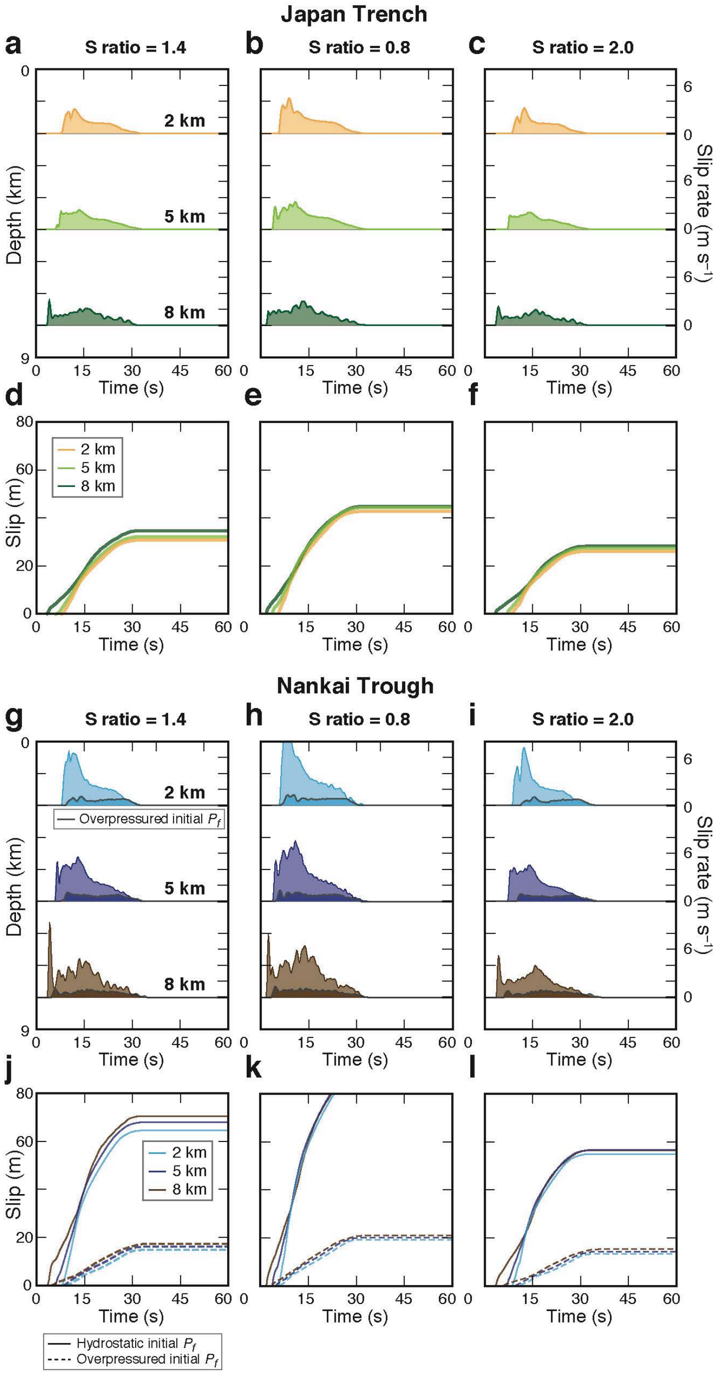

Supplementary Figure S9. Spatio-temporal distribution of near-trench earthquake slip for different S ratios. a–c, Slip rate as a function of time at representative depths (2, 5 and 8 km) along ideal plate-boundary fault with 15° dip in the Japan Trench for various S ratios (**a**, 1.4, **b**, 0.8 and **c**, 2.0). **d–f,** Accumulation of slip with time at each depth and S ratio. **g–i,** Slip rate as a function of time at representative depths (2, 5 and 8 km) along ideal plate-boundary fault with 15° dip in the Nankai Trough for various S ratios (**g**, 1.4, **h**, 0.8 and **i**, 2.0). **j–l,** Accumulation of slip with time at each depth and S ratio. The dashed curves show the results of simulations using the overpressured initial P_f condition.

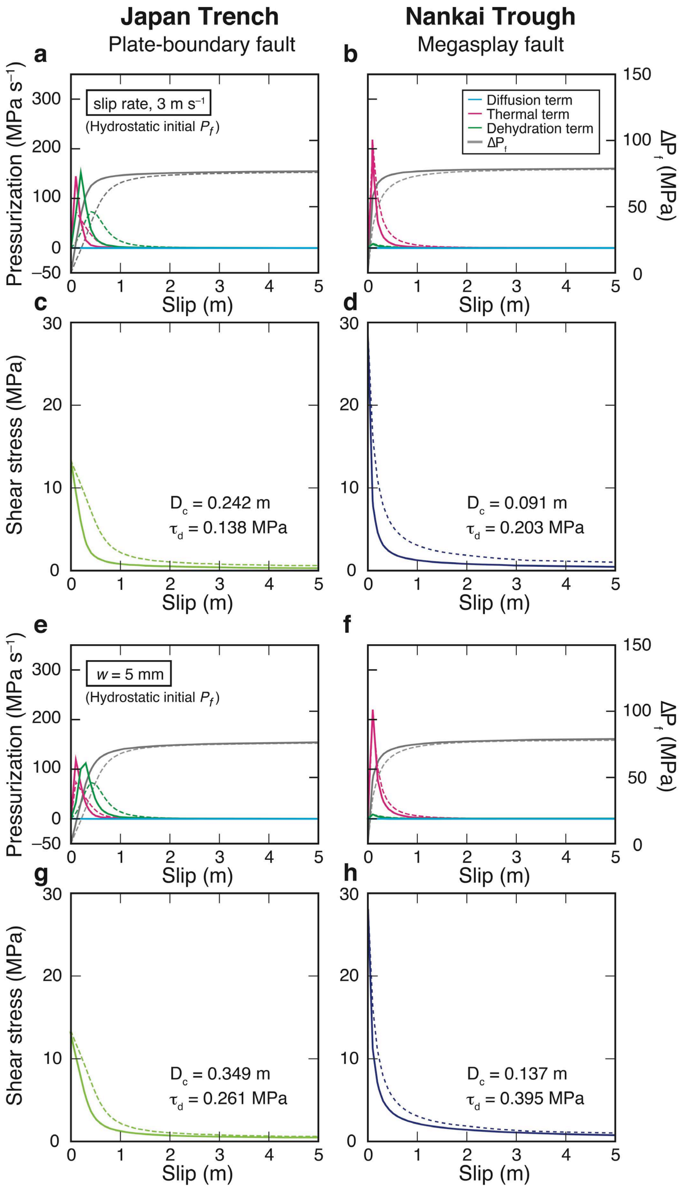

Supplementary Figure S10. Thermal pressurization modelling with a fast slip rate and a thin slip zone under hydrostatic initial P_f condition. a, b, Contributions of the diffusion, thermal, and dehydration terms of equation (1) to the pressurization of interstitial fluid (P_f) at the centre of the slip zone for a slip rate of 3 m s^{-1} at 5 km depth. The dashed lines show the results for a slip rate of 1 m s^{-1} (Fig. 2). **c, d,** Changes of shear stress averaged within the slip zone of the plate-boundary fault of the Japan Trench (left) and of the megasplay fault of the Nankai Trough (right). D_c, critical slip distance; τ_d, dynamic frictional stress. **e, f,** Contribution to the pressurization of interstitial fluid at the centre of a slip zone with a thickness of 5 mm at 5 km depth. The dashed lines show the results for a slip zone with a thickness of 10 mm (Fig. 2). **g, h,** Changes of shear stress averaged within the slip zone. Other parameters used in the TP modelling were set to the same values as those used in the modelling with a 1 m s^{-1} slip rate and a slip zone thickness of 10 mm, described in the main text.

Supplementary Table S1. Original data sheet for the mineral assemblage output by the RockJock program

Selected minerals	J-FAST Scaly clay (821.71–821.73 m) [17R1, 21–23 cm]	J-FAST Scaly clay (822.04–822.06 m) [17R1, 54–56 cm]	J-FAST Scaly clay (822.34–822.35 m) [17R1, 84–85 cm]	J-FAST Scaly clay (822.50–822.51 m) [17R1, 100–101 cm]	J-FAST Scaly clay [Average]	NanTroSEIZE Dark gouge (271.13–271.14 m) [28R2, 22–23 cm]	NanTroSEIZE Scaly clay (271.40–271.42 m) [28R2, 49–51 cm]
Quartz	11.3	13.7	9.0	8.7	10.7	24.8	20.7
Kspar (ordered microcline)	0.0	0.6	0.0	0.0	0.1	0.0	0.0
Kspar (intermediate microcline)	1.9	0.8	0.0	0.0	0.7	1.7	2.4
Kspar (sanidine)	1.4	0.0	3.1	4.0	2.1	0.0	0.6
Kspar (orthoclase)	4.3	6.7	6.1	5.1	5.5	3.2	3.8
Kspar (anorthoclase)	0.0	0.2	0.1	0.4	0.2	0.2	0.0
Plagioclase (albite, var. cleavelandite)	6.6	5.9	3.5	3.0	4.7	9.1	9.9
Plagioclase (oligoclase; NC)	0.0	0.0	0.1	0.0	0.0	3.4	0.0
Plagioclase (oligoclase; Norway)	0.6	0.0	2.0	0.1	0.7	0.2	3.3
Plagioclase (andesine)	2.7	2.9	0.0	0.2	1.5	1.9	1.5
Plagioclase (labradorite)	0.0	0.0	0.8	2.1	0.7	0.0	0.0
Plagioclase (bytownite)	1.8	1.9	0.8	1.3	1.4	3.5	3.8
Plagioclase (anorthite)	1.2	1.2	0.0	0.6	0.8	0.0	0.0
Calcite	0.0	0.0	0.0	0.0	0.0	4.6	4.2
Dolomite	0.0	0.0	0.0	0.0	0.0	0.0	0.3
Ankerite	0.0	0.0	0.0	0.0	0.0	0.0	0.0
Magnesite	0.0	0.0	0.0	0.0	0.0	0.0	0.0
Siderite	0.0	0.0	0.0	0.0	0.0	0.0	0.0
Halite	0.0	0.0	0.0	0.0	0.0	0.7	0.5
Pyrite	0.3	0.1	0.1	0.2	0.2	0.6	0.8
Gypsum	0.0	0.1	0.1	0.0	0.1	0.0	0.0
Magnetite	0.0	0.0	0.1	0.0	0.0	0.0	0.0
Hematite	0.0	0.1	0.0	0.0	0.0	0.0	0.0
Goethite	0.0	0.0	0.6	0.2	0.2	0.0	0.0
Maghemite	0.0	0.0	0.0	0.0	0.0	0.0	0.0
Total non-clay minerals	31.9	34.1	26.3	26.0	29.6	53.9	51.8
Kaolinite (disordered)	0.0	1.3	3.6	4.0	2.3	4.4	3.1
Kaolinite (ordered)	2.4	2.4	0.0	0.0	1.2	0.1	0.0
Smectite (Na-Kinney montmorillonite)	8.1	2.4	11.7	10.1	8.1	0.0	10.4
Smectite (Ca-Kinney montmorillonite)	26.7	26.6	17.2	19.6	22.5	10.8	0.0
Smectite (Saponite)	9.4	7.2	2.9	6.1	6.4	0.6	2.1
Illite (1Md)	0.0	0.0	0.8	0.0	0.2	2.7	8.7
Illite (1M; R>3; 95%I)	0.0	0.9	5.7	6.2	3.2	12.8	1.1
Illite (R>1, 70-80%I)	3.2	3.4	16.2	16.1	9.7	0.5	0.0
Illite (1M; RM30)	1.5	4.8	1.5	0.1	2.0	0.2	2.4
Chlorite (CCa-2)	1.2	1.1	0.0	0.4	0.7	3.9	2.0
Chlorite (CMM)	0.0	0.0	1.0	1.4	0.6	0.8	0.1
Chlorite (Fe-rich; Tusc)	0.0	0.0	0.0	0.6	0.2	0.9	6.7
Chlorite (Mg; Luzenac)	0.4	0.0	0.3	0.0	0.2	0.8	2.0
Muscovite (2M1)	15.2	15.7	12.9	9.5	13.3	7.6	9.6
Total clay minerals	68.1	65.9	73.7	74.0	70.4	46.1	48.2

Supplementary Table S2. Parameters used in the thermal pressurization modelling

	Japan Trench			Nankai Trough		
Parameters		Unit	Remark/ Reference		Unit	Remark/ Reference
Physical propeties						
Density of solid matrix in slip zone	2.46	g cm^{-3}		2.41	g cm^{-3}	
Density of fluid	1.00	g cm^{-3}		1.00	g cm^{-3}	
Bulk density of host rock	2.60	g cm^{-3}		2.60	g cm^{-3}	
Compressibility of fluid	4.30×10^{-10}	Pa^{-1}	ref. 111	4.30×10^{-10}	Pa^{-1}	ref. 111
Friction coefficient (μ)	0.17			0.37		
Environmental conditions						
Normalized pore-pressure ratio	hydrostatic		ref. 17	hydrostatic or 0.8		ref. 18
Geothermal gradient	26.3	°C km^{-1}	ref. 112	37.4	°C km^{-1}	ref. 113
Temperature at seafloor	3.4	°C		2.9	°C	
Thickness of slip zone (w)	0.01	m		0.01	m	
Slip velocity	1.0	m s^{-1}		1.0	m s^{-1}	
Hydraulic properties						
Initial porosity	$93.037 \times P_{e0}^{-0.37725}$		P_{e0}, initial effective pressure (Pa)	$3.1612 \times P_{e0}^{-0.12476}$		P_{e0}, initial effective pressure (Pa)
Porosity-P_e equation	porosity = b × P_e^{c}		P_e , effective pressure (Pa)	porosity = b × P_e^{c}		P_e , effective pressure (Pa)
Constant b	ϕ_0/P_{e0}^{c}		ϕ_0, initial porosity	ϕ_0/P_{e0}^{c}		
Constant c	-0.1541			-0.27623		
Initial permeability	$2.6239 \times 10^{-22} \times \exp(14.926 \times \phi_0)$	m^2		$0.00047799 \times P_{e0}^{-2.0562}$	m^2	
Permeability-P_e equation	$2.6239 \times 10^{-22} \times \exp(14.926 \times \phi)$	m^2	$\phi = b \times P_e^{c}$	$d \times P_e^{e}$	m^2	
Constant d	-			k_0/P_{e0}^{e}		k_0, initial permeability
Constant e	-			-0.02473		
Viscosity of fluid	$2.414 \times 10^{-5} \times 10^{(247.8/(T + 133))}$	Pa s	ref. 114	$2.414 \times 10^{-5} \times 10^{(247.8/(T + 133))}$	Pa s	ref. 114
Thermal properties						
Specific heat capacity of solid matrix	1.00	J g^{-1} K^{-1}		0.85	J g^{-1} K^{-1}	
Specific heat capacity of fluid	4.20	J g^{-1} K^{-1}		4.20	J g^{-1} K^{-1}	
Thermal diffusivity of solid matrix	6.0×10^{-7}	m^2 s^{-1}	ref. 115	6.0×10^{-7}	m^2 s^{-1}	ref. 115
Thermal diffusivity of fluid	1.7×10^{-7}	m^2 s^{-1}		1.7×10^{-7}	m^2 s^{-1}	
Coefficient of thermal expansion of solid matrix	2.20×10^{-5}	K^{-1}	ref. 116 for quartz	2.20×10^{-5}	K^{-1}	ref. 116 for quartz
Coefficient of thermal expansion of fluid	5.00×10^{-4}	K^{-1}	ref. 111	5.00×10^{-4}	K^{-1}	ref. 111
Chemical kinetics						
A_{p_1st}	4.27×10^{4}	s^{-1}		3.47×10^{-1}	s^{-1}	
E_{a_1st}	52.1	kJ mol^{-1}		27.7	kJ mol^{-1}	
Kinetic function of 1st-step dehydration	F3			D3		
Enthalpy of 1st-step dehydration	30.2	J g^{-1}		16.9	J g^{-1}	
Weight loss of 1st-step dehydration	3.10	weight %		1.66	weight %	
Volume loss of 1st-step dehydration	0.987			0.993		
A_{p_2st}	6.46×10^{4}	s^{-1}		4.47×10^{2}	s^{-1}	
E_{a_2st}	114.0	kJ mol^{-1}		80.7	kJ mol^{-1}	
Kinetic function of 2nd-step dehydration	An (n=0.5)			F3		
Enthalpy of 2nd-step dehydration	195.5	J g^{-1}		152.2	J g^{-1}	
Weight loss of 2nd-step dehydration	4.30	weight %		5.80	weight %	
Volume loss of 2nd-step dehydration	0.982			0.976		

*Normalised pore pressure ratio = (pore pressure - hydrostatic pressure) / (confining pressure - hydrostatic pressure)

A_p; pre-exponential term; E_a, activation energy; An, n-dimensional nucleation; F3, third-order reaction

Supplementary Table S3. Parameters used with the slip-weakening friction law at each depth under hydrostatic initial P_f condition

Location	Depth (km)	Initial shear stress (MPa)	Yield shear stress (MPa)	Dynamic frictional stress (MPa)	Critical slip distance (m)	Correlation coefficient
Japan Trench	1	1.098	2.667	0.265	3.949	0.996
	2	2.197	5.335	0.289	1.861	0.993
	3	3.295	8.003	0.297	1.153	0.991
	4	4.394	10.670	0.295	0.800	0.990
	5	5.492	13.338	0.290	0.589	0.990
	6	6.590	16.005	0.292	0.449	0.990
	7	7.689	18.673	0.286	0.351	0.990
	8	8.787	21.340	0.276	0.277	0.990
	9	9.885	24.007	0.268	0.221	0.990
	10	10.984	26.675	0.267	0.176	0.990
Nankai Trough	1	2.416	5.806	0.623	0.963	0.933
	2	4.833	11.612	0.524	0.545	0.936
	3	7.249	17.417	0.494	0.380	0.944
	4	9.666	23.223	0.471	0.296	0.952
	5	12.082	29.029	0.455	0.247	0.956
	6	14.499	34.835	0.433	0.214	0.963
	7	16.915	40.640	0.417	0.190	0.949
	8	19.332	46.446	0.402	0.172	0.953
	9	21.748	52.252	0.381	0.159	0.956
	10	24.164	58.057	0.361	0.148	0.960

Supplementary Table S4. Parameters used with the slip-weakening friction law at each depth under overpressured initial P_f condition

Location	Depth (km)	Initial shear stress (MPa)	Yield shear stress (MPa)	Dynamic frictional stress (MPa)	Critical slip distance (m)	Correlation coefficient
Nankai Trough	1	0.483	1.161	0.182	3.145	0.975
	2	0.967	2.322	0.375	2.380	0.960
	3	1.450	3.484	0.607	1.572	0.929
	4	1.933	4.645	0.558	1.269	0.930
	5	2.416	5.806	0.514	1.050	0.931
	6	2.900	6.967	0.476	0.889	0.932
	7	3.383	8.128	0.443	0.766	0.933
	8	3.866	9.289	0.414	0.671	0.934
	9	4.350	10.450	0.388	0.596	0.935
	10	4.833	11.611	0.365	0.535	0.937

Supplementary References:

111. Fine, R. A. & Millero, F. J. Compressibility of water as a function of temperature and pressure. *J. Chem. Phys.* **59,** 5529–5536 (1973).

112. Fulton, P. M. *et al.* Low coseismic friction on the Tohoku-Oki fault determined from temperature measurements. *Science* **342**, 1214–1217 (2013).

113. Sugiura, T. *et al.* Re-evaluation of temperature at the updip limit of locked portion of Nankai megasplay inferred from IODP Site C0002 temperature observatory. *Earth Planets Space* **66**:107 (2014).

114. Fontaine, F. J., Rabinowicz, M. & Boulegue, J. Permeability changes due to mineral diagenesis in fractured crust; Implications for hydrothermal circulation at mid-ocean ridges. *Earth Planet. Sci. Lett.* **184,** 407–425 (2001).

115. Hirono, T. & Hamada, Y. Specific heat capacity and thermal diffusivity and their temperature dependencies in a rock sample from adjacent to the Taiwan Chelungpu fault. *J. Geophys. Res.* **115**, B05313 (2010).

116. Bayer, G. Thermal expansion anisotropy of oxide compounds. *Proc. Br. Ceram. Soc.* **22**, 39–53 (1973).
